# Development and performance assessment of an advanced Lucas‐Kanade algorithm for dose mapping of cervical cancer external radiotherapy and brachytherapy plans

**DOI:** 10.1002/acm2.13249

**Published:** 2021-05-04

**Authors:** Václav Novák, Jaroslav Ptáček, Petr Fiala, Zuzana Vlachová, Paulina Jašková

**Affiliations:** ^1^ Department of Medical Physics and Radiation Protection University Hospital Olomouc Czech Republic; ^2^ Department of Nuclear Medicine University Hospital Olomouc Czech Republic; ^3^ Department of Oncology University Hospital Olomouc Czech Republic

**Keywords:** deformable image registration, Lucas‐Kanade algorithm, treatment planning

## Abstract

**Purpose:**

The aim of this study was to verify the possibility of summing the dose distributions of combined radiotherapeutic treatment of cervical cancer using the extended Lucas‐Kanade algorithm for deformable image registration.

**Materials and methods:**

First, a deformable registration of planning computed tomography images for the external radiotherapy and brachytherapy treatment of 10 patients with different parameter settings of the Lucas‐Kanade algorithm was performed. By evaluating the registered data using landmarks distance, root mean square error of Hounsfield units and 2D gamma analysis, the optimal parameter values were found. Next, with another group of 10 patients, the accuracy of the dose mapping of the optimized Lucas‐Kanade algorithm was assessed and compared with Horn‐Schunck and modified Demons algorithms using dose differences at landmarks.

**Results:**

The best results of the Lucas‐Kanade deformable registration were achieved for two pyramid levels in combination with a window size of 3 voxels. With this registration setting, the average landmarks distance was 2.35 mm, the RMSE was the smallest and the average gamma score reached a value of 86.7%. The mean dose difference at the landmarks after mapping the external radiotherapy and brachytherapy dose distributions was 1.33 Gy. A statistically significant difference was observed on comparing the Lucas‐Kanade method with the Horn‐Schunck and Demons algorithms, where after the deformable registration, the average difference in dose was 1.60 Gy (*P*‐value: 0.0055) and 1.69 Gy (*P*‐value: 0.0012), respectively.

**Conclusion:**

Lucas‐Kanade deformable registration can lead to a more accurate model of dose accumulation and provide a more realistic idea of the dose distribution.

## 1 INTRODUCTION

Locally advanced cervical cancer is standardly treated using a combination of concomitant chemotherapy, external radiotherapy (EBRT), and brachytherapy (BRT) boost to the cervical region.^1^

The current standard for dose accumulation of the combined radiotherapeutic treatment according to the International Commission on Radiation Units and Measurements (ICRU) report 89 is based on the simple DVH parameter addition without employing an adequate registration model.^2^ Doses for absolute organs at risk (OAR) volumes are estimated by adding dose‐volume histogram (DVH) parameters from each fraction, assuming that the location of a given hotspot volume (e.g., 2 cm^3^) is identical in each fraction.^3^ So the worst alternative should always be considered. For critical organs, this indicates that the maximum dose calculated in each fraction is always realized in the same volume of tissue. On the contrary, in the case of tumors, the minimum doses always meet in the same volume.

Changes in the patient's irradiation position between EBRT and BRT and the introduction of applicators close to the tumor before each fraction of BRT causes organ movement and soft tissue deformation. As a result, the specific tissue structures generally occupy a completely new position compared to previously applied radiation fields. The tissue structures bring previously accumulated doses to these new positions, which are often significantly different from those that would correspond to their current position. To correctly express the accumulated dose, the absorbed doses must be summed up not in the same spatial coordinates, but always in the same anatomical volume of tissue.^4^

Therefore, a prerequisite for proper adaptive dose accumulation planning is the registration of shifts that occur within the tissues between individual fractions. These displacements are formally described by the deformation field. Appropriate anatomy imaging is the starting point for determining shifts between fractions. The general registration scheme involves the selection of a reference fraction, to which so‐called floating images, corresponding to the remaining fractions of treatment, are subsequently related. Using a reference and a floating image, an appropriate registration algorithm can then be employed to determine the deformation fields. Knowledge of the deformation field enables reconstruction of the actual position of the tissues with respect to the applied radiation fields, thus, determining the appropriate contribution to the accumulated dose in each fraction. This procedure is referred to as dose mapping. The use of the concept of dose mapping by image registration thus, enables the addition of doses in corresponding anatomical areas, even when applied in different fractions of combined radiotherapy.

The concept described above was used in this study to develop a software (SW) tool designed to optimize dose prescription in combined cervical radiotherapy. The application of such a tool to real tissue changes is merely an approximation of reality. Therefore, an integral part of the design of a model tool for dose mapping using image registration is careful testing of its statements.

Thus, this work aimed to create an SW tool containing an algorithm for deformable image registration (DIR) and to verify its possibilities for adaptive planning of combined EBRT and BRT treatment of patients with cervical cancer.

## 2 MATERIALS AND METHODS

The study was divided into two parts. The setting of the parameters of the algorithm for the registration of computed tomography (CT) images and dose mapping of the combined EBRT and BRT was specified in retrospective study #1. The optimization phase of study #1, in which the optimal parameters of the registration algorithm were assessed, was performed on a group of 10 patients with cervical cancer. The second part of this study (study #2) assessed the performance of the algorithm by evaluating the accuracy of dose mapping on 10 other patients and compared the results with the current standard of dose accumulation in combined radiotherapy.

The fractionation parameters applied in the treatment of patients whose CTs were used in this study were EBRT 45 Gy in 25 fractions on planning target volume including nodes and then boost to the cervical region 6 Gy in three fractions using the volumetric modulated arc therapy (VMAT). After completing an external radiotherapy course, patients were treated with 28 Gy intracavitary BRT on high‐risk clinical target volume in four fractions using a Fletcher Utrecht CT compatible applicator.

Compared to conventional radiotherapeutic procedures, combined radiotherapy is characterized by significantly different fractionation parameters during external and brachytherapeutic parts. For this case, ICRU report 89 recommends recalculating the absorbed dose distribution to the equieffective dose. The equieffective dose related to normofractionation is denoted by the symbol EQD2.^2^, ^5^ The following relationship was used to calculate EQD2 based on the applied doses:

(1) $$EQD2=D∙\left(\frac{\frac{\alpha }{\beta }+d}{\frac{\alpha }{\beta }+2}\right),$$

in which the ratio α/β was set to 10 Gy for tumor response and 3 Gy for late effect in critical organs.

The registration scheme was chosen so that the planning CT image acquired for the last fraction of the BRT application served as a reference image, and the CT study created to plan EBRT served as a floating image.

CT images were created using a GE LightSpeed RT 16 scanner (GE Healthcare, Waukesha, USA) with a slice thickness of 2.5 mm. Individual CT slices together with files containing dose distribution and contours of structures of interest were exported from planning systems Monaco v.3.3, (Elekta AB, Stockholm, Sweden) and Oncentra v.4.3 (Elekta AB, Stockholm, Sweden) in the Digital Imaging and Communications in Medicine (DICOM) format. All other operations with these files, including deformable registration, evaluation, dose mapping, and conversion to EQD2 and dose volume histogram calculations, were then performed in MATLAB R2013a (The Mathworks Inc., Massachusetts, US).

### 2.A Image preprocessing

The presence of an applicator in the reference image represents a major problem when registering floating images, taken as a part of EBRT planning, to a reference image corresponding to the BRT. In general, it is not possible to register a part of the data that is present in a single image. As per the procedure suggested by Moulton et al,^6^ before the application of the deformable registration, preparatory steps were made to modify each BRT study.

1. Individual Fletcher applicator catheters were manually contoured in each CT slice and replaced with random Hounsfield units (HU) values in the range of +/‐200 HU around the mean HU value of the surrounding tissue.
2. The replaced area was blurred using a Gaussian filter.

### 2.B Rigid and affine preregistration

Application of the algorithm for deformable image registration to significantly different CT studies of individual fractions of combined radiotherapy demonstrated that for its effective functioning, it is advantageous to first preregister all CT studies manually using rigid transformation to match the corresponding bone structures. Improved image data matching was further achieved by using a relatively fast automatic affine preregistration of floating images to the reference image. The result of these steps was then followed by deformable registration to fine‐tune the image similarity.

### 2.C Lucas‐Kanade deformable registration

Considering the results of studies^7^, ^8^, ^9^, ^10^ comparing the accuracy of different approaches for deformable CT data registration, the Lucas‐Kanade (LK) algorithm was used. This method works with the concept of deformation as a continuous optical flow between CTs. The results of the above studies indicate that when modeling “physiological” deformations in the pelvic or thoracic region, methods based on the concept of optical flow show comparable accuracy to other algorithms, whether commercially or freely available. The LK method was extended by the pyramid representation of the image and the implementation of weighted windows in the least‐squares method. This creates the prerequisites for its smooth use in the area of extreme deformations, which are characteristic in BRT, and to further increase the accuracy of registration results.

The LK algorithm is based on the assumption that the optical flow is approximately constant in a small neighborhood (or window) of the considered point.^11^ Under these conditions, exactly one equation generating the dynamics of the optical flow based on the other points can be constructed for each point of the selected neighborhood. Consideration of all surrounding points leads to a system of linear equations whose solution is the optical flow for the considered point. Thus, the size of the neighborhood determines the number of equations. Generally, it is preferable to assign a higher weight in each equation to points that are closer to the considered point, thus using a weighted version of the least‐squares method.^12^, ^13^

#### 2.C.1 Iterative calculation of optical flow by pyramid version of Lucas‐Kanade method

**Fig. 1:**
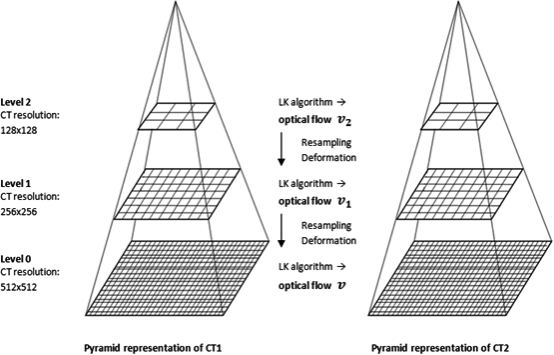
Determination of optical flow by the pyramid extension of the Lucak‐Kanade method: CT studies are registered first at the highest level of the image pyramid with the lowest resolution. At this level, the optical flow is calculated using the LK algorithm. The result of this calculation is then extended to a lower level in the form of an initial estimate. At the lower level, the residual optical flow is then calculated. In this way, it gradually continues to lower levels with higher resolution (finer details), which allows to register even large deformations while maintaining the condition of small movement.

Using this extension of the LK method, a CT study is represented by an image pyramid. Each level of the pyramid consists of the same CT, differing only in resolution. The lowest level represents CT slices with the highest resolution, given by the imaging modality (512 × 512 pixels in the case of CT). Higher levels then contain images with half the resolution of the previous one. Thus, the image with the lowest resolution is at the top of the pyramid.^14^ The optical flow calculation algorithm is illustrated in Fig. 1.

### 2.D Patient study #1 – registration parameters setting

The function of the selected registration algorithm can be further optimized for a specific application. The accuracy of image data registration is essential for the subsequent mapping of dose distributions of individual patients, as the same deformation vector field is used.

In study #1, the values and combinations of the number of image pyramid levels and size of the neighborhood around a given point were optimized.

Various metrics have been proposed to calculate the similarity of images and to assess registration accuracy. However, when evaluating registration errors under real conditions, it appears that none of the available criteria can be applied universally, and the application of a single criterion often leads to incorrect conclusions. An example might be the failure of image similarity evaluation procedures based on image intensity in areas of homogeneous intensity.^15^ Regarding the recommendations of publications^6^ and,^16^ a combination of the following approaches was used to assess the accuracy of registration when optimizing the parameters.

#### 2.D.1 Landmarks distance

In the landmark‐tracking technique, the degree of similarity is determined by the distance between the corresponding points marked in both the floating and reference images. When testing the algorithm, at least 100 well‐recognizable matching points were manually identified in both CT studies by an experienced physician. After registration, the average 3D distance for each patient was calculated.

Since the area to which the BRT boost dose distribution was to be delivered was smaller than the area of interest for the EBRT, the selection of landmarks was not uniform across the CT study. A denser network of points (a minimum of five points in a given CT slice) was marked in slices affected by the BRT dose distribution. In other parts of the CT covered only by the EBRT dose, a smaller number of points were marked (always a minimum of two points in the slice).

#### 2.D.2 Mean squared error (MSE)

This approach is based on the comparison of the distribution of HU in the corresponding images using a certain mathematical or statistical criterion. The sum of squares of HU differences at the individual points of CT studies was used as an image similarity metric. Owing to the use of different positioning aids and the different patient's position on the CT table when acquiring the planning CT for the EBRT and BRT fractions, the MSE was evaluated only within the patient.^6^ The registration error is then listed in the results section as the root mean squared error (RMSE).

#### 2.D.3 Gamma analysis

MSE‐based evaluation can produce significant errors in steep changes in intensity, even in the case of very small registration inaccuracies. Landmark distance measurement, in turn, does not provide any information on movement in areas where no marks are selected; therefore, proper deformation in these areas is not guaranteed. Thus, a more robust method of comparing two intensity distributions was used, which, to some extent, combines both approaches and considers not only differences in intensity (or HU) at a given point but also spatial differences such as gamma analysis.^17^, ^18^

Gamma analysis was performed only on the patient's contour. For practical reasons, Verisoft v.7.1 software (PTW, Freiburg, Germany) was used to evaluate the agreement between individual CT slices by two‐dimensional gamma analysis. The evaluation criteria were set to 2%/2 mm with reference to local “dose.” An average gamma score specifying the percentage of image pixels that fulfilled the selected criteria, was determined for the given registration parameters.

### 2.E Patient study #2‐ DIR‐based dose accumulation

#### 2.E.1 Evaluation and comparison of dose accumulation accuracy

Evaluation of dose accumulation accuracy was performed on a group of 10 other patients with cervical cancer receiving combined treatment (patient study #2). In both CT studies for EBRT planning and the last fraction of BRT boost, more than 100 well‐recognized, corresponding points of interest (POIs) were manually marked by an experienced physician. The equieffective dose values were measured at these points. The deformation vector fields calculated within the CT image registration using the LK method with optimized parameters found in study #1 were used to deform the dose distributions and their subsequent summation. The cumulative dose values obtained from the landmarks in the reference image were then compared with the sum of the doses at the corresponding points before registration. The degree of agreement was then expressed as an average of the absolute and relative dose differences at individual landmarks for each patient.

BRT applications are characterized by a steep dose distribution gradient, and even a small positional error in image registration can cause large dose variations at a given point. For this reason, the dose at landmarks was calculated as the average of the doses from six surrounding points.

To verify and compare the results of the optimized LK algorithm, CT studies of these 10 patients were subsequently registered using two other algorithms, based on the intensity of the image with the sum of the square of intensity difference metric – Horn‐Schunck (HS) method and a method using a modified Demons algorithm. Both algorithms are part of the freely available software, DirArt.^19^, ^20^ The results of registrations using these independent algorithms were imported into the developed SW tool. Considering the conclusions of published studies,^6^, ^7^, ^9^, ^10^ indicating that both methods were sufficiently accurate in comparison with other freely or commercially available algorithms, the results were used as a reference to evaluate the accuracy of the tested registration model.

#### 2.E.2 Comparison with the current standard of dose accumulation

After mapping the dose distributions, the results can be presented as a summary DVH of EBRT and BRT treatment expressed in EQD2.

GEC‐ESTRO Gynaecology group^21^ recommends to report dose‐volume values in the high‐dose region – the minimum EQD2 to the 0.1 cm^3^ (D_0.1cm_^3^) and 2 cm^3^ (D_2cm_^3^) volumes of the OAR that receive the maximum dose. To compare DIR‐based dose accumulation from the study#2 with the current standard of dose accumulation (which, after conversion to EQD2, provides for the simple sum of DVHs for the dose parameters) differences in D_0.1cm_^3^ and D_2cm_^3^ of bladder for each patient were found. These parameters were obtained from DVHs calculated for the EBRT course and the last BRT fraction.

#### 2.E.3 Statistical methods

Welch’s two‐sample *t* test was used to compare the average dose differences at landmarks between the LK algorithm and the HS and Demons method across all 10 cases from study #2. *P*‐values <0.05 were considered statistically significant. Statistical analysis was performed using RStudio version 1.0.153 (RStudio, Boston, MA, USA).

## 3 RESULTS

An algorithm for deformable registration of CT images, the pyramid version of the LK method with a weighted window, was created in MATLAB. This software also includes scripts enabling rigid and affine preregistration, deformation of dose distribution and contour shapes, determination of registration accuracy based on the distance of corresponding points and RMSE of image intensities, and assessment of the dose mapping accuracy by calculating the dose differences at landmarks.

### 3.A Patient study #1 ‐ finding optimal registration parameters

Tables 1, 2, 3 show the accuracy of registration using a modified LK algorithm for various parameter settings according to the individual evaluation criteria.

**Table 1:** Evaluation of the Lucas‐Kanade (LK) registration results by measuring the distance of landmarks for various settings of LK registration parameters.

Patient	Average landmarks distance [*mm*] (range)					
	After affine registration	LK pyramid level 2			LK pyramid level 3	
		Neighborhood radius 2	Neighborhood radius 3	Neighborhood radius 4	Neighborhood radius 2	Neighborhood radius 3
1	3.45 (0.2‐7.8)	1.91 (0.1‐4.1)	1.70 (0.1‐6.3)	2.43 (0.1‐5.9)	2.73 (0.1‐6.3)	2.45 (0.2‐5.7)
2	4.17 (0.1‐9.6)	2.34 (0.3‐7.4)	1.68 (0.0‐4.9)	2.12 (0.3‐6.7)	2.80 (0.2‐6.6)	2.37 (0.2‐7.7)
3	5.01 (0.2‐13.1)	2.05 (0.1‐4.1)	1.98 (0.1‐5.3)	2.85 (0.2‐9.9)	4.10 (0.1‐7.5)	3.10 (0.1‐7.2)
4	4.43 (0.3‐11.3)	2.78 (0.3‐6.6)	2.53 (0.2‐6.3)	2.52 (0.1‐5.9)	3.93 (0.2‐7.4)	3.69 (0.3‐11.1)
5	9.05 (0.4‐22.4)	5.76 (0.3‐15.7)	5.34 (0.1‐13.0)	6.50 (0.1‐16.3)	7.05 (0.1‐12.5)	6.35 (0.4‐14.6)
6	5.10 (0.1‐15.1)	3.32 (0.1‐8.7)	1.96 (0.1‐5.3)	3.03 (0.1‐9.8)	4.07 (0.1‐11.4)	3.38 (0‐10.9)
7	5.56 (0.1‐12.7)	1.88 (0.2‐4.7)	1.50 (0.2‐5.2)	1.98 (0‐4.8)	3.08 (0.1‐12.4)	3.22 (0.2‐8.5)
8	4.60 (0.2‐12.9)	2.25 (0.1‐4.2)	2.55 (0.1‐9.1)	2.97 (0.1‐8.2)	4.05 (0.1‐16.1)	3.20 (0.3‐8.6)
9	4.93 (0.3‐13.3)	1.93 (0.1‐6.2)	2.24 (0.2‐7.7)	2.81 (0.2‐5.9)	3.79 (0.2‐9.6)	2.81 (0.1‐7.1)
10	4.73 (0.1‐10.9)	2.48 (0‐6.3)	2.04 (0.1‐7.3)	2.33 (0.1‐5.4)	3.33 (0.1‐10.1)	2.88 (0.1‐8.1)
Combined distance (SD)	5.10 (3.83)	2.67 (2.53)	2.35 (2.18)	2.95 (2.76)	3.89 (3.17)	3.35 (2.72)

The table shows the average values of the measured distance, including their range (minimum‐maximum measured distance) for the individual patient. The combined distance denotes the average distance for all patients. All values are expressed in mm.

Abbreviation: SD, standard deviation.

**Table 2:** The root mean squared error (RMSE) of intensities of registered images for various settings of the Lucas‐Kanade (LK) registration parameters.

Patient	RMSE					
	After affine registration	LK pyramid level 2			LK pyramid level 3	
		Neighborhood radius 2	Neighborhood radius 3	Neighborhood radius 4	Neighborhood radius 2	**Neighborhood radius 3**
1	181.01	69.16	66.83	71.76	99.72	91.89
2	184.22	68.44	68.21	68.41	102.43	91.51
3	202.9	85.35	79.30	86.07	110.97	95.70
4	200.37	87.08	80.96	88.04	112.15	95.78
5	234.15	132.13	127.46	135.18	145.27	137.39
6	204.31	88.87	82.78	87.43	106.01	102.79
7	190.97	71.99	73.64	71.92	100.07	99.08
8	193.20	84.00	83.08	86.36	117.74	107.62
9	189.32	78.19	77.82	78.73	103.35	96.28
10	186.72	81.23	76.98	81.84	106.05	97.51
Average	196.72	84.64	81.71	85.57	110.38	101.56

**Table 3:** Comparison of the Lucas‐Kanade (LK) registration results by 2D gamma analysis of individual slices.

Patient	Average gamma score [%]					
	After affine registration	LK pyramid level 2			LK pyramid level 3	
		Neighborhood radius 2	Neighborhood radius 3	Neighborhood radius 4	Neighborhood radius 2	Neighborhood radius 3
1	68.1	88.7	90.4	88.4	77.3	81.1
2	67.2	89.3	90.2	89.8	75.9	79.1
3	67.1	86.1	88.0	85.9	74.2	79.5
4	64.2	85.1	88.6	85.2	74.1	78.8
5	49.7	66.8	67.5	64.4	59.0	63.7
6	66.4	86.3	86.1	85.6	76.8	77.7
7	69.9	88.4	91.4	88.7	78.8	81.8
8	65.5	84.0	87.5	86.8	72.4	78.6
9	65.7	86.3	89.3	86.7	77.7	80.7
10	66.7	87.1	88.3	86.3	75.9	79.0
Average	65.1	84.8	86.7	84.8	74.2	78.0

The values in the table give the average gamma score for each patient for different settings of the LK registration parameter.

#### 3.A.1 Landmarks distance

Table 1 lists the average landmark distances for the different settings of the LK algorithm parameters for both individual patients and the combined average distance (average of all points for all patients) for more than 1100 landmarks for all patients. The average registration error ranged from 2.35 mm (for setting two pyramid levels and the radius of a neighborhood of 3 voxels) to 3.89 mm (for three pyramid levels and the neighborhood radius of 2 voxels). Considering this evaluation, the measured distance of landmarks appears to increase with a higher number of pyramid levels.

#### 3.A.2 Mean squared error

Table 2 summarizes the results of the image similarity evaluation using the RMSE. Unlike measuring the landmark distance, RMSE assesses the similarity in each voxel of the CT study and not just in a preselected sample. However, the results are quite similar to those of landmark evaluation. According to the RMSE, the best final agreement was again achieved by a combination of registration parameters: two pyramid levels and the radius of the neighborhood of 3 voxels.

**Fig. 2:**
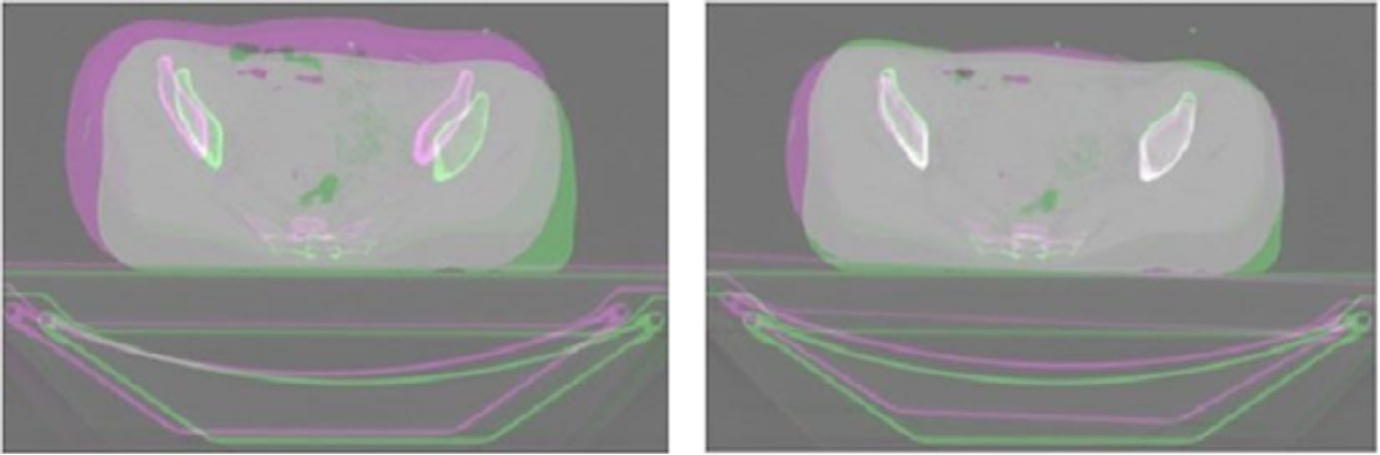
Difference between CT studies of patient no. 1 before registration (left) and after affine registration (right).

**Fig. 3:**
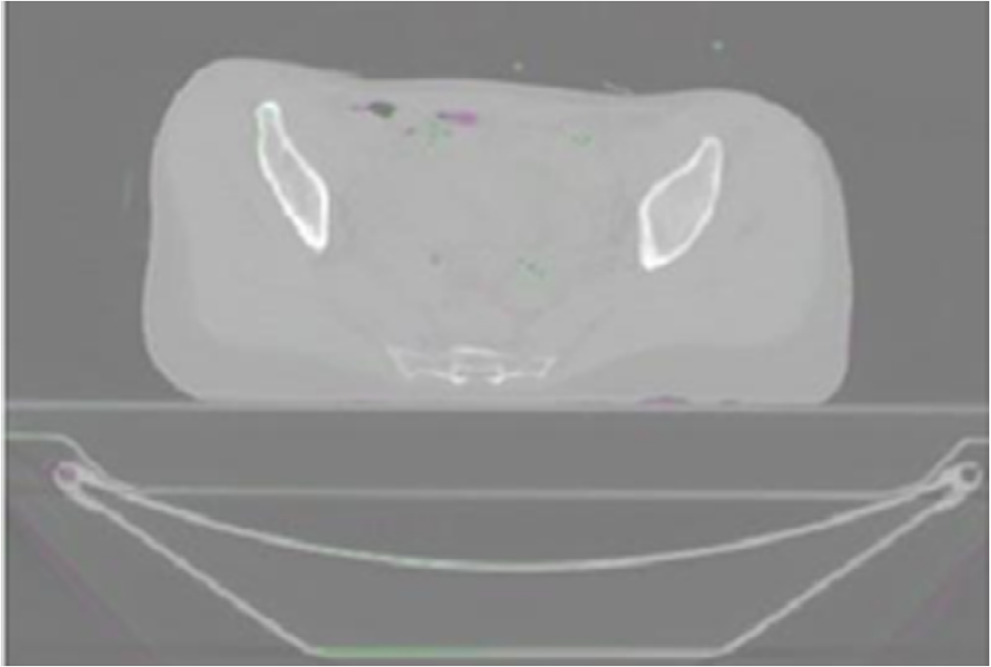
Difference between CT studies of patient no. 1 after deformable registration by optimized pyramid LK method with weighted window (pyramid level 2, neighborhood radius of 3 voxels).

Figures 2 and 3 show the differences between CT studies created for EBRT and BRT planning of patient no. 1, each after the individual steps of their mutual registration. Better results of the final agreement of both CT studies were achieved using affine preregistration, which was performed before the final application of the LK algorithm.

#### 3.A.3 Gamma analysis

The evaluation of the image similarity by gamma analysis is presented in Table 3 and confirms the results of the previous methods. When setting the gamma analysis parameters to 2mm/2%, the highest mean gamma score of registered CT studies of all patients was 86.7%.

#### 3.A.4 Evaluation summary

The results of the evaluation using all three selected criteria correspond to each other. Based on the average landmark distance, RMSE, and gamma scores, the best agreement was achieved for two pyramid levels and a neighborhood radius of 3 voxels. The parameters optimized in this manner were introduced into the LK algorithm and further used for deformable registration in patient study #2.

**Fig. 4:**
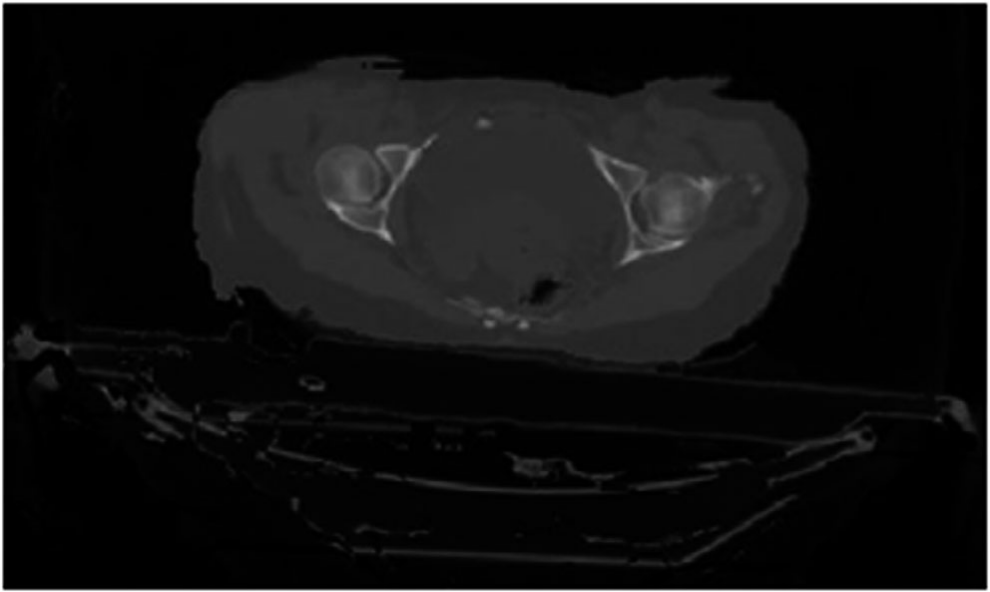
Example of error in CT deformable registration.

Regarding patient no. 5 (in Tables 1, 2, 3), the average distance of the corresponding points was doubled compared to the others, and the average gamma score after affine preregistration was <50%. Probably due to the significantly different distribution of the patient's tissues during the acquisition of the planning CT for BRT and EBRT, the presented SW model failed. Moreover, its application generated visually unrealistic deformations (Fig. 4). It should also be noted that no form of post‐optimization regularization was used in the method.

### 3.B Patient study #2 ‐ DIR‐based dose accumulation

#### 3.B.1 Evaluation and comparison of dose accumulation accuracy

Table 4 summarizes the mean absolute and relative dose differences in POIs for 10 patients after affine and LK image registration. The table also includes the corresponding values calculated by the HS and modified Demons algorithm.

**Table 4:** Average absolute and relative differences of equieffective doses at landmarks for individual patients after the Lucas‐Kanade (LK) deformable registration, including comparison with the Horn‐Schunck and Demons algorithm for both individual patients and a summary of average differences for all patients (combined dose difference).

Patient	Average EQD2 difference in POI (range)							
	After affine registration		Lucas‐Kanade		Horn‐Schunck		Demons	
	Abs [Gy]	Rel [%]	Abs [Gy]	Rel [%]	Abs [Gy]	Rel [%]	Abs [Gy]	Rel [%]
1	2.93 (0‐11.9)	5.23 (0.1‐9.3)	1.42 (0.1‐5.7)	2.65 (0.2‐42.4)	1.80 (0.1‐6.6)	2.82 (0.1‐8.3)	1.75 (0‐5.8)	2.64 (0‐11.3)
2	3.08 (0‐9.4)	5.31 (0.9‐10.1)	1.44 (0‐5.4)	2.70 (0‐20.5)	1.77 (0‐5.8)	2.99 (0.2‐11.1)	1.92 (0‐7.2)	2.37 (0.2‐6.6)
3	3.02 (0.1‐11.8)	4.91 (0.1‐13.6)	1.38 (0‐6.9)	2.32 (0‐9.4)	1.43 (0.1‐5.6)	2.41 (0.1‐10.4)	1.71 (0.1‐6.4)	2.39 (0‐7.4)
4	2.89 (0‐10.3)	4.27 (0‐10.8)	1.28 (0‐4.7)	2.02 (0‐14.1)	1.52 (0.1‐5)	2.28 (0.1‐5.4)	1.71 (0.1‐4.7)	2.41 (0‐8.5)
5	2.32 (0‐10.9)	3.86 (0.2‐23.9)	1.06 (0.1‐7.2)	1.78 (0.1‐7.8)	1.11 (0‐6.5)	2.20 (0.1‐8.2)	1.21 (0.1‐7)	2.25 (0‐11.7)
6	3.40 (0.1‐13.8)	5.48 (0.1‐53.7)	1.31 (0‐5.3)	2.44 (0.2‐11.3)	1.58 (0.1‐8.1)	2.45 (0‐14.8)	1.42 (0‐6.8)	2.34 (0‐10.4)
7	2.58 (0.1‐9.6)	4.89 (0.2‐22.4)	1.24 (0‐4.2)	2.36 (0‐27.9)	1.59 (0‐6.5)	2.81 (0.1‐10.1)	1.68 (0‐4.3)	2.65 (0.1‐10.9)
8	3.44 (0‐19)	5.11 (0.2‐30.7)	1.19 (0‐7.6)	2.08 (0.1‐8.2)	1.66 (0‐12.6)	2.77 (0.1‐8.7)	1.59 (0.1‐6.4)	3.02 (0.1‐12.6)
9	3.06 (0.2‐10.6)	5.19 (0.1‐33.8)	1.65 (0‐7.7)	3.14 (0.3‐7.1)	1.79 (0.1‐7)	3.33 (0.1‐9.2)	2.01 (0.1‐5.5)	3.21 (0‐10.1)
10	3.29 (0.1‐10)	5.01 (0‐20.1)	1.34 (0.1‐7.2)	3.21 (0.1‐18.7)	1.72 (0‐4.9)	3.41 (0‐13.6)	1.88 (0.1‐5.3)	3.30 (0.1‐10.6)
Combined dose distance	3.00	4.93	1.33	2.47	1.60	2.75	1.69	2.66

Abbreviation: POI, point of interest.

The statistical analysis revealed a significant difference in the average dose differences at landmarks between the extended LK algorithm and HS (*P* = 0.0055) and modified Demons (*P* = 0.0012) algorithm. Therefore, the accuracy of the LK method appears to be slightly higher than that of others.

#### 3.B.2 Comparison with the current standard of dose accumulation

**Fig. 5:**
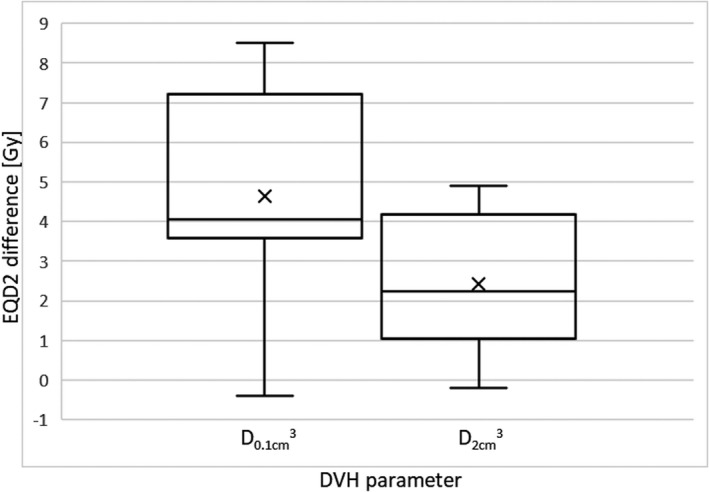
EQD2 differences between bladder DVH parameters obtained from simple DVH parameter addition and DIR‐based dose accumulation.

Figure 5 shows the differences in D_0.1cm_^3^ and D_2cm_^3^ (expressed in EQD2) for bladder between simple DVH parameter addition and DIR‐based dose accumulation. Mean dose deviation was 4.6 ± 2.6 Gy (9.6 ± 5.9%) and 2.4 ± 1.7 Gy (5.3 ± 3.9%) for D_0.1cm_^3^ and D_2cm_^3^ relative to DIR, respectively. The ranges of the dose deviations were −0.4‐8.5 Gy and −0.2‐4.9 Gy (−0.8‐20.6% and −0.4‐11.2% relative to DIR) for D_0.1cm_^3^ and D_2cm_^3^, respectively. These values show that the DVH parameter addition is likely to overestimate the reported dose‐volume parameters for bladder in combined radiotherapeutic treatment compared to the method based on the LK deformable registration.

## 4 DISCUSSION

The presented results indicate that the proposed SW tool, using the LK algorithm extended by the implementation of weighted windows and pyramidal representation of the image, can be used to estimate the cumulative dose of combined EBRT and BRT with higher accuracy than similar approaches.^7^

The accuracy of the deformable registration was significantly influenced by parameter settings. The selection of the optimal level of the pyramid iteration and size of the neighborhood was made based on the evaluation of the registration accuracy for various settings. The unambiguity of the final selection underlines the fact that optimization led to similar values for all evaluation methods.

The results of patient study #1 show that the best agreement was reached for a low number of pyramid levels. This is consistent with the experience that for significant tissue shifts or deformations in the patient's body, requiring larger apertures, the reliability of the registration decreases. For the same reason, the registration error also increases with the increasing size of the neighborhood around the considered point. For pyramid level 2 and the radius of the neighborhood of 4 voxels (results not included in Tables 1, 2, 3), inaccuracies begin to arise on the body surface, where the algorithm is more sensitive to steep changes in contrast. In the case of LK registration with pyramid level 3 and the radius of the neighborhood of 4 voxels, the entire volume already showed visually unrealistic deformations in all evaluated patients (similar to Fig. 4).

The accuracy evaluation of deformable registration reported in Tables 1, 2, 3 characterizes only the overall agreement of the registered CT study with the reference one. However, important information also provides a detailed assessment of the accuracy of registration for individual CT slices. As expected, in this way of evaluation, significant errors were found, particularly in outer CT slices, that is, outside the region of interest of radiotherapy. The gamma scores often dropped below 50%. From this, it can be concluded that in the area of interest, approximately in the middle of the CT study, the agreement evaluated by gamma analysis was better than the stated average of 86.7%.

Kadoya et al^7^ compared several types of algorithms for the deformable registration of CT data in chest study. For the case of HS and modified Demons methods, the authors reported the combined distance of landmarks after registration of 2.4 mm and 2.42 mm, respectively. In their opinion, the achieved accuracy provides the potential for the application of these methods in clinical practice. If the distance calculated using the HS and Demons algorithm can be considered a criterion of the potential of the registration method for practical use, then the results of our study #1 (combined landmark distance of 2.35 mm) support the implementation of the extended LK method in practice.

In patient study #2, the accuracy of dose deformation and mapping of dose distributions using the LK algorithm was evaluated. According to statistical analysis, the results summarized in Table 4 demonstrate a significant difference in the average dose difference at landmarks between the LK method and HS and modified Demons algorithms included in the DirArt software. A certain drawback is that low‐dose areas were not distinguished from high‐dose areas when calculating dose differences. A small difference in absolute dose could thus represent a large difference in the relative dose and vice versa. To reduce this side effect at least partially, the relative differences were calculated with respect to the global maximum dose.

Finally, a comparison of the dose‐volume parameters of bladder between the DIR‐based dose accumulation and the simple DVH parameter addition for EBRT planning dose and the last BRT fraction was made. Similar to studies,^3^, ^22^ also results of this study show higher mean dose values of D_0.1cm_^3^ and D_2cm_^3^ for bladder for the current standard of dose accumulation. Especially D_0.1cm_^3^ seems to be less robust to this approximation with a mean deviation of 9.6 ± 5.9% as compared to DIR. As for the D_2cm_^3^, simple DVH parameter addition provides an estimate with a mean deviation of 5.3 ± 3.9% relative to DIR. Unlike this study, the authors in 3 compared dose accumulation for two BRT fractions and got lower deviations in D_0.1cm_^3^ and D_2cm_^3^ of 5.2 ± 4.2% and 1.5 ± 1.8%, respectively. This was to be expected as larger deformations may occur when registering EBRT to BRT.

In addition to the possible advantages of the presented algorithm, the results of this work also showed the limits of its use.

Although the results indicate the potential use of the method to generate an accumulated dose estimate with the stated accuracy, its practical use significantly limits the need for manual removal of the applicator introduced into the patient's body in BRT applications. This procedure could also introduce an additional error into the evaluation process.

Furthermore, it appears that the algorithm fails in cases of extreme changes in the position of the tissues between the floating and reference CT images. Unfortunately, these cases are not exceptional when EBRT is combined with BRT (results for patient no. 5 in Tables 1, 2, 3). In this context, it should be noted that the higher robustness of the presented tool against extreme deformations could be provided mainly by the addition of the LK algorithm with a pyramidal representation of the CT image. However, the pyramid approach is not limited in this regard.

Thus, the use of manual alignment of bone structures and affine preregistration of CT images is of similar importance to the inclusion of the pyramid method. Without using these auxiliary steps, the results for the entire patient data tested were erroneous, as shown in Fig. 4.

**Fig. 6:**
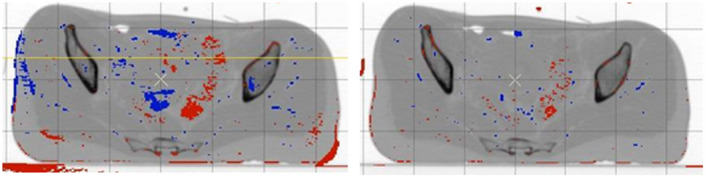
Comparison of the agreement of individual CT slice of patient no. 1 using gamma analysis after affine registration (left) and after deformable registration by LK with optimized parameters (right) (pyramid level 2, neighborhood radius of 3 voxels).

**Fig. 7:**
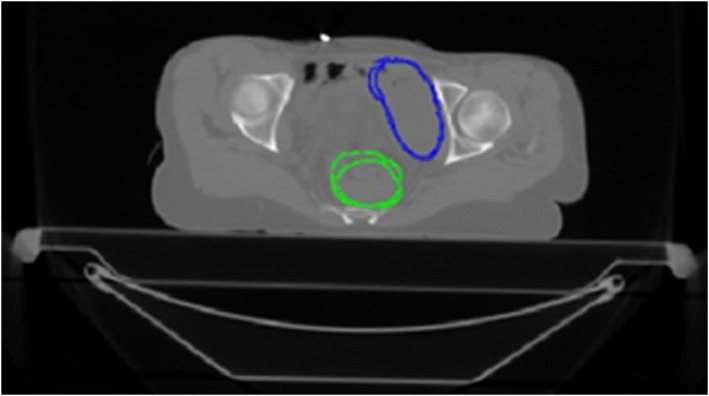
Rectum (green) and bladder (blue) matching differences for patient no. 1 of study #1.

Registration inaccuracies were more pronounced, particularly in the case of soft tissues. Bone structures were usually paired correctly owing to the different densities. In Fig. 6, which shows the results of the comparison by gamma analysis, it can be observed that in the case of soft tissues in areas with homogeneous image intensity, the detected registration errors were higher. This is a relatively serious finding as the registration errors are subsequently transmitted to the deformation of the contours of critical structures, such as the bladder and rectum (Fig. 7).

## 5 CONCLUSION

This study shows that the extended LK method of DIR could provide competitive accuracy of dose accumulation. The properly deformed and summed dose distribution after each BRT fraction could then be used for adaptive planning of the following fraction.

Based on the comparison with the current standard of dose accumulation, it can be concluded that the use of deformable registration should allow radiation oncologists to gain a more realistic view of the dose distribution within the patient.

The outcomes of this study also indicate some possibilities for further development of procedures for the preliminary detection of CT studies unsuitable for this type of registration; that is, finding a criterion for the magnitude of the anatomical change between fractions in which DIR fails.

## CONFLICT OF INTEREST

No conflict of interest.

## AUTHOR CONTRIBUTIONS

Study concept and design: Václav Novák. Software programming: Vaclav Novak, Petr Fiala, Jaroslav Ptáček. Clinical revision: Zuzana Vlachová. Statistical analysis: Paulina Jašková.

## Data Availability

Data available from the corresponding author upon request.
